# Pandemics and Income Inequality: What Do the Data Tell for the Globalization Era?

**DOI:** 10.3389/fpubh.2021.674729

**Published:** 2021-05-28

**Authors:** Tiejun Chen, Giray Gozgor, Chun Kwong Koo

**Affiliations:** ^1^School of Management, Zhejiang University of Technology, Hangzhou, China; ^2^Faculty of Political Sciences, Istanbul Medeniyet University, Istanbul, Turkey; ^3^HeXie Management Research Centre, Xi'an Jiaotong-Liverpool University, Suzhou, China

**Keywords:** COVID-19 crisis, pandemics uncertainty, World Pandemic Discussion Index, WPDI, Income inequality, Feasible General Least Squares estimations

## Abstract

This paper empirically investigates the effects of pandemics uncertainty on income inequality We consider a new measure of pandemics uncertainty, the World Pandemic Discussion Index (WPDI), and the post-tax (net) Gini coefficient We focus on the panel data of 141 countries from 1996 to 2020. The results from the Feasible General Least Squares estimations indicate that the WPDI is negatively related to income inequality in 107 non-OECD countries. However, the WPDI is positively associated with income inequality in 34 OECD economies. This evidence remains robust when considering different models, including several controls, and implementing various sensitivity analyses.

## Introduction

The COVID-19 pandemic again shows us that there can be widespread negative economic effects of a global pandemic (1–3). At this stage, there are various papers on how the COVID-19 pandemic has affected economic and financial indicators. For example, Bakas and Triantafyllou (4) observe that the COVID-19 pandemic has increased commodity price volatility. Chakrabarty and Roy (5) show the positive effects of the COVID-19 pandemic on fiscal stimulus. Gupta et al. (6) state that the COVID-19 pandemic has slowed down the world's macroeconomic activity. Wu (7) indicates that the pandemics-related uncertainty has negatively affected household consumption across the countries.

On the other hand, the COVID-19 pandemic is expected to significantly affect income inequality (8). There are various indicators to determine income inequality, including demographics, economic performance, globalization, government policies, institutions' quality, and labor market regulations (especially unions) [see e.g., (9–16)]. Given these backdrops, this paper aims to investigate the effects of pandemics uncertainty (measured by the World Pandemic Discussion Index-WPDI) and the income inequality [measured by the post-tax (net) Gini coefficient] in the panel dataset of 141 countries from 1996 to 2020.

There are various channels (hypotheses) on how pandemics can affect income inequality. The impact can be positive or negative. The first channel is the direct effect on the mortality rate. The Spanish Flu in 1918 mostly affected young men, and there was a direct impact of this pandemic on labor supply and labor income (1). However, the pandemics in the 21st century, including the COVID-19, have mostly affected older people. Therefore, there are negligible impacts of these pandemics on labor income. The first channel of pandemics can decrease income inequality in developing economies.

The second channel is the decline of households' income and the rise of precautionary savings. According to World Bank (17), the lock-down policies and the isolation measures during the COVID-19 pandemic caused a decline in the households' income. This issue leads to lower household consumption and increased precautionary savings. Wu (7) also confirms this hypothesis by showing that pandemics have reduced household consumption in the panel data of 138 countries from 1996 to 2017. Note that an increase in the precautionary savings can also hurt returns on the capital (3, 18), affecting income inequality. The second channel of pandemics can also reduce income inequality in developing economies.

The third channel is fiscal policy. During the COVID-19 crisis, governments introduced fiscal stimulus packages to increase their credibility and to mitigate the pandemics' negative effects on households and the real economy (19). However, these fiscal stimulus packages will lead to a rise in public debts or tax rates in the forthcoming years. Indeed, previous papers have shown that the tax policies' changes or rising public debts significantly affect income inequality (14, 20, 21). The third channel of pandemics can increase income inequality in developed economies.

However, some developed countries, such as Japan, have considerably high public debts to keep public policies associated with a social-democratic welfare state. Many countries have resources to provide credit, minimum income and social welfare policies. The experiences in China and Vietnam, i.e., the systems of market socialism, characterized by the combination of market freedom in some markets and central planning state, have generated successful results for the pandemics. Overall, there are mainstream economic hypotheses and heterodox neo-Keynesian economics to analyze the effects of pandemics on income inequality.

We hypothesize that there are negative effects of pandemics on income inequality in developing economies; ceteris paribus, the impact is positive in developed countries according to the three channels.

There are a few papers in the literature on how pandemics have affected income inequality. For instance, Sayed and Peng (22) use the Fixed-effects and the Augmented Mean Group estimators to examine pandemics' effects on income inequality globally (mainly based on France, Germany, the United Kingdom, and the United States) from 1915 to 2017. The authors find that there is a suppressing impact of pandemics on income inequality. However, the channels on how pandemics can decrease income inequality remain unclear. Galletta and Giommoni (8) find that the Spanish flu pandemic in 1918 significantly and persistently increased the income inequality in the Italian municipalities. This evidence comes from the issue that there is a significant decline in poor people's income share. However, Alfani (23) and Alfani and Ammannati (24) observe that the 14th-century Black Death plague decreased the Italian regions' income inequality in the following centuries. There is also mixed evidence on the effects of different pandemics on income inequality in the Italian regions during the different pandemics. Using the unbalanced panel dataset of 175 countries from 1961 to 2017; Furceri et al. (25) find that pandemics have caused an increase in the Gini coefficient and higher-income deciles' income shares. Furceri et al.'s (25) approach is based on the shocks of dummy variables for pandemics. Our paper uses the WPDI; therefore, we can measure and compare the uncertainty due to the pandemic's magnitude over time across the countries.

We attempt to contribute to the related empirical literature by investigating the effects of pandemics uncertainty on income inequality. Our paper examines the effects of pandemics uncertainty, measured by a new index, so-called WPDI, and the income inequality, measured by the post-tax Gini coefficient, in the panel data of 141 countries over the period 1996–2020. The WPDI indicator is based on country reports, focusing on pandemic-related events, policy uncertainty, and policy implications on pandemics. The WPDI indicator is similar to business cycle fluctuations, and it has a significant impact on income via pandemic-related uncertainty shocks (26). Therefore, we suggest that the WPDI should have similar income effects with the uncertainty mechanism is valid in the previous literature [see e.g., (27–31)].

At this point, we contribute to the current empirical literature on the relationship between pandemics and inequality by addressing several issues. First, we use different models to tackle potential reverse causality. There are several findings to observe the opposite direction in the COVID-19 era, i.e., inequality affects the virus's spread. According to Ahmed et al. (32), poor people lack access to health services, and they are vulnerable during times of economic crisis. Simultaneously, the less-educated workers have fewer remote works opportunities (33). Therefore, poor workers should go to their work, usually by the public transport system, increasing the virus transmission. Overall, the virus can spread at a higher level in countries where income inequality is a serious problem, such as the United States (34). By using the lagged right-side variables, we address a possible reverse causality issue. Note that the pandemic-related events and pandemics uncertainty is purely exogenous (26). In other words, the WPDI will not theoretically be affected by income inequality.

Secondly, there could be omitted variable bias given that the determinants of income inequality are complex. This paper includes various control variables to capture the effects of demographics, globalization, government size, institutions' quality, labor market conditions, and macroeconomic conditions on income inequality.

Thirdly, we split the countries as the OECD and the non-OECD countries to address the countries' case at different economic development stages. This empirical examination has been very useful since we have observed the mixed effects of pandemics on income inequality in different countries.

Finally, we implement various sensitivity analyses to check the robustness of the findings. For instance, we exclude the countries with extreme inequality levels and extreme uncertainty related to the pandemics. Thus, we show that outliers do not drive the results. Besides, we exclude the countries in different regions, such as East Asia (where the pandemic has been under control since the very begging) and Latin America (it has been the most fragile region regarding the new type of coronavirus). Our results indicate that the WPDI is negatively related to income inequality in 107 non-OECD countries. However, the WPDI is positively associated with income inequality in 34 OECD economies from 1996 to 2020.

The rest of the paper is organized as follows. Section Empirical Model, Methodology, and Data describes the data and empirical models and explains the estimation procedures. Section Empirical Results presents the empirical findings. Section Robustness Checks provides the robustness checks. Section Conclusion concludes.

## Empirical Model, Methodology, and Data

### Empirical Model and Estimation Procedure

We estimate the following equations:

(1) $$\begin{array}{r} Inequality_{i,t}=\alpha_{0}+\alpha_{1}\;WPDI_{i,t}+{\alpha_{2}\;X}_{i,t}+\;\vartheta_{t}+{\vartheta_{i}+\varepsilon }_{i,t} \end{array}$$

(2) $$\begin{array}{r} Inequality_{i,t}=\beta_{0}+\beta_{1}\;WPDI_{i,t-1}+{\beta_{2}\;X}_{i,t}+\;\vartheta_{t}+{\vartheta_{i}+\varepsilon }_{i,t} \end{array}$$

(3) $$\begin{array}{r} Inequality_{i,t}=\gamma_{0}+\gamma_{1}\;WPDI_{i,t}+{\gamma_{2}\;X}_{i,t-1}+\;\vartheta_{t}+{\vartheta_{i}+\varepsilon }_{i,t} \end{array}$$

(4) $$\begin{array}{r} Inequality_{i,t}=\delta_{0}+\delta_{1}\;WPDI_{i,t-1}+{\delta_{2}\;X}_{i,t-1}+\;\vartheta_{t}+{\vartheta_{i}+\varepsilon }_{i,t} \end{array}$$

In Equations from (1) to (4), *Inequality*_*i,t*_ is the current income inequality, based on the Gini index for the post-tax income levels in country *i* at time *t*. *WPDI*_*i,t*_ and *WPDI*_*i,t*−1_ are the current and the lagged World Pandemic Discussion Index in country *i* at time *t* and *t-k*. *X*_*i,t*_ and *X*_*i,t*−1_ are current and the lagged vector of controls. Finally, ϑ_*t*_, ϑ_*i*_, and ε_*i,t*_ indicate “time random-effects,” “country random-effects,” and the “error term,” respectively. We hypothesize that there are negative effects of pandemics on income inequality in developing economies; ceteris paribus, the impact is positive in developed countries according to the three channels discussed in the introduction.

We estimate these equations using the Feasible General Least Squares (FGLS), the common estimator in the empirical literature [see, e.g., (7, 35–37)].

### Data

Our dataset includes the annual frequency panel data from 1996 to 2020 in 141 countries. Note that the WPDI data are available until 2020Q4. Still, the inequality and other measures data are merely available until 2019 at best. Therefore, we use the forecast values of the income inequality and other indicators to capture the effects of the COVID-19 pandemic in 2020. Since we aim to focus on the countries at different development stages, we also split the countries as the non-OECD economies (107 countries) and OECD economies (34 countries) in the dataset. We use the annual-frequency data to capture the effects of business cycles on income inequality. We report a list of countries in our dataset in Appendix. Specifically, we consider the following variables in the estimations.

#### Dependent Variable

Following previous papers [e.g., (25)], we use the post-tax (net) Gini coefficients to measure income inequality. It is an index from 0 to 100. We obtain the related data from the Standardized World Income Inequality Database (SWIID) (version 9.1) of Solt (38).

#### World Pandemic Discussion Index

Our research's novelty is that to use a new pandemics uncertainty measure, so-called the WPDI (26). This indicator is based on the text-mining of the country reports in the Economist Intelligence Unit (EIU). At this stage, we focus on the country-level indices on the discussion about pandemics. These indices are constructed by counting the number of times a word related to pandemics is mentioned in the EUI country reports. Ahir et al. (26) consider the following keywords in the EUI country reports: Severe Acute Respiratory Syndrome, SARS, Avian Flu, H5N1, Swine Flu, H1N1, Middle East Respiratory Syndrome, MERS, Bird Flu, Ebola, Coronavirus, COVID-19, Influenza, H1V1, World Health Organization, and WHO. These indices are the percent of the words related to the above pandemics-related words in the EIU country reports, multiplied by 1,000. A greater value means greater discussion, thus uncertainty about pandemics (26). For details, refer to https://worlduncertaintyindex.com/data/. We expect that there can be negative effects of pandemics on income inequality in developing economies; however, the impact should be positive in developed countries according to the three channels discussed in the introduction.

**Figure 1:**
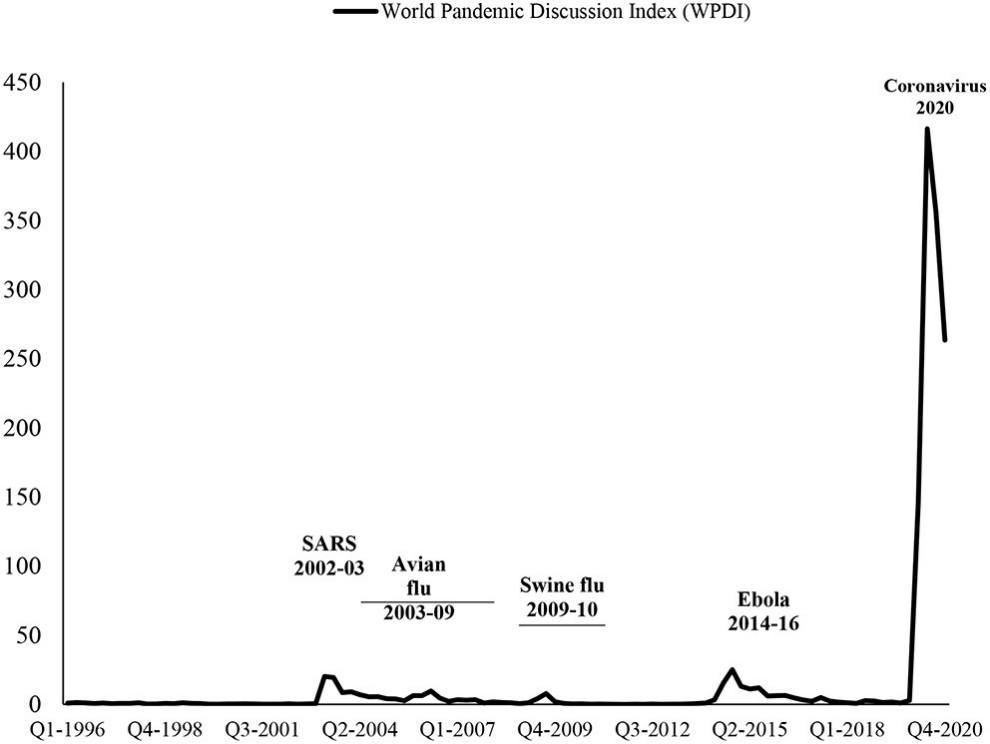
World Pandemic Discussion Index (WPDI) (1996–2020). Data Source: Ahir et al. (26): https://worlduncertaintyindex.com/data/.

The sample considered in our paper starts in 1996. Some events, such as the Severe Acute Respiratory Syndrome (SARS) in 2002–2003, Avian Flu (H5N1) in 2003–2009, Swine Flu (H1N1) in 2009–2010, Middle East Respiratory Syndrome (MERS) in 2014–2020, Bird Flu in 2013–2017, Ebola in 2014–2016, Coronavirus (COVID-19) in 2020-ongoing, lead to rising pandemics-related uncertainty in the globe, as it is presented in Figure 1.

Figure 1 indicates that the WPDI has little trend; until the COVID-19, it does not change significantly over time. However, Figure 1 provides the WPDI at the global level. There are significant variations in the level of the WPDI across the countries, given that most of these pandemics remain at the regional level rather than the global level. It is also important to note that the WPDI is driven by fully unpredictable shocks, significantly affecting income inequality. For instance, Gupta et al. (6) find that increases in the global pandemics-related uncertainty index are related to the future slowdowns in the global gross domestic product (GDP) growth. Therefore, the WPDI should also be a leading countercyclical variable, affecting income inequality across the countries. Therefore, we also use the lagged WDPI to avoid a possible issue the reverse causality. Furthermore, we observe no reverse causality issue when we run a formal test of panel causality.

#### Control Variables

Following the empirical approach in Gozgor and Ranjan (12), we include the per capita GDP in the constant $ prices to capture income level and the age-dependency ratio (% of working-age population) to control demographics and trans-generational spillover of the income. According to the Kuznets Curve hypothesis (13), the per capita income decreases income inequality in developed countries (OECD countries in our case). It increases income inequality in developing countries (non-OECD countries in our case). The age dependency ratio should be positively related to income inequality since this issue hurts wealth distribution against poor people. These data come from the World Development Indicators dataset in World Bank (39).

We also add various additional controls to check the robustness of the benchmark findings. As additional controls, we first use the total unemployment rate to control macroeconomic conditions. We expect that unemployment is positively related to income inequality.

Secondly, we consider total population (in logarithmic form) and the urban population relative to total population to capture the effects of demographics on the cross-country differences in income inequality. Generally, total population and urban populations increase income inequality due to the spatial concentration of economic activities.

Thirdly, we add female labor force participation rate and labor market regulations (an index from 0 to 10) to control labor market indicators in the estimations. All of these indicators are obtained from World Bank (39), except for the labor market regulations index, which is obtained from the Economic Freedom of the World Dataset (version 2020), provided by Gwartney et al. (40). Female labor force participation should be negatively linked to income inequality. Freer labor market regulations can increase income inequality, according to the previous empirical papers.

Fourthly, we include control variables to capture government size in the economy via the index from 1 to 10 and the share of transfer and subsidies relative to the GDP. We obtain these data from Gwartney et al. (40). Higher transfers and subsidies are expected to decrease income inequality.

Fifthly, pandemics can affect income inequality through globalization level channels, which may escalate to the pandemics-related uncertainty (41) or directly affect income inequality (12). At this stage, we add the revised version of the KOF indices of globalization (version 2020) for the KOF overall globalization index, introduced by Gygli et al. (42). For the details of the KOF indices of globalization, also refer to Gozgor (43), Dreher (44), and Potrafke (45). Globalization can increase income inequality since it promotes capital gains and decreases the relative income of labor (12).

Sixthly, we control institutions' quality since formal institutions can change the effects of pandemics shocks on income inequality (25). Therefore, we use the democracy/autocracy spectrum, measured by the Revised Combined Polity Score (Polity2) (an index from −10 to +10). These data are obtained from the Polity V Annual Time Series, introduced by Marshall and Gurr (46). Higher quality of institutions can increase the power of the welfare state; thus, it should decrease income inequality.

Finally, details of the variables and a summary of descriptive statistics are provided in Table 1.

**Table 1:** Descriptive statistics.

**Indicator**	**Definition**	**Data source**	**Mean**	**Standard deviation**	**Minimum**	**Maximum**	**Obs**.
Post-tax Gini coefficient	Index from 0 to 100	(38 )	39.10	8.578	22.11	67.55	2,857
World Pandemic Discussion Index	Index	(26)	3.036	17.55	0.000	438.9	3,408
Per capita GDP (constant US$)	Logarithmic form	(39)	8.340	1.545	5.233	11.43	3,377
Age dependency ratio	% of Working-age population	(39)	63.59	19.58	15.74	114.2	3,400
Female labor force participation rate	% of female population ages 15+	(39)	51.74	16.45	5.831	87.68	3,408
Total population	Logarithmic form	(39)	16.44	1.351	13.16	21.05	3,400
Total unemployment rate	% of total labor force	(39)	7.761	6.029	0.090	37.98	3,408
Urban population	% of total population	(39)	56.38	22.92	7.410	100.0	3,400
Transfers and subsidies	Share of GDP	(40)	9.064	7.835	0.000	34.10	2,643
Government size	Index from 0 to 10	(40)	6.504	1.427	0.120	9.510	3,113
Labor market regulation	Index from 0 to 10	(40)	6.107	1.436	2.100	9.730	2,565
Overall globalization	Index from 0 to 100	(42)	59.06	15.93	22.53	90.98	3,266
Democracy/autocracy spectrum	Index from −10 to 10	(46)	3.635	6.301	−10.00	10.00	3,216

## Empirical Results

### Results of the Model With Non-lagged Controls

Table 2 provides the results of the FGLS estimations for the models in Equations (1, 2) from 1996 to 2020. The dependent variable is the post-tax Gini coefficient.

**Table 2:** Feasible General Least Squares (FGLS) (non-lagged controls) (1996–2020).

**Sample**	**All countries**	**All countries**	**Non-OECD**	**Non-OECD**	**OECD**	**OECD**
**Regressor**	**(1)**	**(2)**	**(3)**	**(4)**	**(5)**	**(6)**
Log per capita GDP*_*t*_*	−1.951^***^ (0.048)	−1.998^***^ (0.048)	2.307^***^ (0.051)	2.278^***^ (0.051)	−6.133^***^ (0.187)	−6.275^***^ (0.180)
Age Dependency*_*t*_*	0.092^***^ (0.003)	0.087^***^ (0.003)	0.214^***^ (0.003)	0.213^***^ (0.003)	0.355^***^ (0.022)	0.360^***^ (0.022)
WPDI*_*t*_*	−0.010^**^ (0.004)	–	−0.002^**^ (0.001)	–	0.069^***^ (0.020)	–
WPDI_t−1_	–	−0.006 (0.004)	–	−0.002 (0.001)	–	0.070^***^ (0.020)
Intercept	49.48^***^ (0.608)	50.24^***^ (0.612)	9.612^***^ (0.573)	9.970^***^ (0.582)	76.55^***^ (2.291)	77.86^***^ (2.219)
Observation	2,801	2,669	1,991	1,889	810	780
Countries	141	141	107	107	34	34
Wald Test [Probability]	10,285^***^ [0.000]	9,845^***^ [0.000]	5,529^***^ [0.000]	5,464^***^ [0.000]	1,370^***^ [0.000]	1,505^***^ [0.000]

*The dependent variable is the post-tax Gini coefficient_t_. The standard errors are in ().*

*** *p < 0.01 and*

** *p < 0.05*.

In the entire sample, the estimated coefficients of the current WPDI and the lagged WPDI are −0.010 and −0.006, respectively. However, only the current WPDI is statistically significant at the 5% level (see Columns 1 and 2, Table 2). The findings for 107 non-OECD countries are reported in Columns 3 and 4, while the results for 34 OECD countries are provided in Columns 5 and 6. The effect of the WPDI on income inequality is also adverse in non-OECD countries. Similarly, only the coefficient of the current WPDI is statistically significant at the 5% level.

Interestingly, the impact of the WPDI on income inequality is positive in the OECD countries, and both the estimated coefficients of the current WPDI and the lagged WPDI are statistically significant at the 1% level. This evidence shows that pandemics-related uncertainty has different effects on income inequality in developed countries to developing countries. The negative impact of pandemics-uncertainty shocks on income inequality can also be related to informal income sources in developing countries. Developing countries' income may not be sensitive to the uncertainty, which is measured by the WPDI.

When we analyze the controls, the per capita GDP is positively related to income inequality in the non-OECD countries. However, the per capita income is negatively associated with income inequality in the OECD countries and the entire sample. This evidence is consistent with the Kuznets Curve discussions, indicating a positive relationship between per capita income and income inequality at the first economic development stage. The income inequality will be decreased as per capita income increases (13). Besides, the age dependency ratio positively affects income inequality in all groups of countries. All of the related coefficients are statistically significant at the 1% level. Finally, the Wald test statistics show that the models are valid (*p* < 0.001).

### Results of the Model With Lagged Controls

Table 3 reports the FGLS estimations' findings for the models in Equations (3, 4) from 1996 to 2020. Again, the dependent variable is the post-tax Gini coefficient.

**Table 3:** Feasible General Least Squares (FGLS) estimations (lagged controls) (1996–2020).

**Sample**	**All countries**	**All countries**	**Non-OECD**	**Non-OECD**	**OECD**	**OECD**
**Regressor**	**(1)**	**(2)**	**(3)**	**(4)**	**(5)**	**(6)**
Log per capita GDP_t−1_	−1.864^***^ (0.048)	−1.861^***^ (0.048)	2.265^***^ (0.052)	2.265^***^ (0.053)	−6.057^***^ (0.189)	−6.062^***^ (0.189)
Age Dependency_t−1_	0.100^***^ (0.003)	0.100^***^ (0.004)	0.211^***^ (0.003)	0.211^***^ (0.003)	0.375^***^ (0.022)	0.375^***^ (0.023)
WPDI*_*t*_*	−0.009^**^ (0.004)	–	−0.004^***^ (0.001)	–	0.067^***^ (0.020)	–
WPDI_t−1_	–	−0.006 (0.004)	–	−0.003^**^ (0.001)	–	0.068^***^ (0.020)
Intercept	48.27^***^ (0.612)	48.23^***^ (0.612)	10.09^***^ (0.594)	10.08^***^ (0.598)	74.83^***^ (2.358)	74.84^***^ (2.359)
Observation	2,669	2,669	1,889	1,889	780	780
Countries	141	141	107	107	34	34
Wald Test [Probability]	10,059^***^ [0.000]	10,018^***^ [0.000]	5,230^***^ [0.000]	5,217^***^ [0.000]	1,365^***^ [0.000]	1,370^***^ [0.000]

*The dependent variable is the post-tax Gini coefficient_t_. The standard errors are in (). *

*** *p < 0.01 and*

** *p < 0.05*.

The Wald test statistics show that all models are valid (*p* < 0.001). In the entire panel data sample, the estimated coefficients of the current WPDI and the lagged WPDI are −0.009 and −0.007, respectively. At this point, the current WPDI is significant at the 5% level (see Column 1, Table 3). Similarly, the results for 107 non-OECD countries are provided in Columns 3 and 4, while the findings for 34 OECD countries are reported in Columns 5 and 6. The WPDI significantly decreases the income inequality in the non-OECD countries, and the related coefficients are statistically significant at the 5% level at least.

Furthermore, the effect of the WPDI on income inequality is positive in the OECD countries. Note that the estimated coefficients of the current WPDI and the lagged WPDI are statistically significant at the 1% level. This evidence confirms the previous findings in Table 3; that is, pandemics-related uncertainty has different effects on income inequality in developed and developing countries.

Looking at the control variables, we observe that the per capita GDP is positively associated with income inequality in non-OECD countries. However, the per capita income is negatively related to the income inequality in the OECD economies and the full sample. Again, this evidence is consistent with the Kuznets Curve hypothesis. Furthermore, the age dependency ratio increases the income inequality in all countries, and this evidence is also in line with the theoretical expectations. These coefficients are statistically significant at the 1% level.

## Robustness Checks

### Robustness to the Inclusion of Other Controls

Tables 4–6 report the findings of robustness to the inclusion of several additional controls for the lagged control model with the current WUI in Equation (3) for the post-tax income inequality using the data from 1996 to 2020 in all countries, non-OECD economies, and OECD economies.

**Table 4:** Feasible General Least Squares (FGLS) estimations (all countries) (1996–2020).

**Sample**	**All countries**	**All countries**	**All countries**	**All countries**	**All countries**	**All countries**	**All countries**	**All countries**	**All countries**
**Regressor**	**(1)**	**(2)**	**(3)**	**(4)**	**(5)**	**(6)**	**(7)**	**(8)**	**(9)**
Log per capita GDP_t−1_	−1.912^***^ (0.044)	−1.978^***^ (0.045)	−1.786^***^ (0.040)	−2.251^***^ (0.052)	−0.175^**^ (0.069)	−1.766^***^ (0.058)	−2.759^***^ (0.044)	−0.660^***^ (0.005)	−2.137^***^ (0.047)
Age dependency_t−1_	0.107^***^ (0.003)	0.087^***^ (0.003)	0.116^***^ (0.003)	0.213^***^ (0.003)	0.070^***^ (0.004)	0.124^***^ (0.005)	0.072^***^ (0.004)	0.081^***^ (0.003)	0.101^***^ (0.004)
WPDI*_*t*_*	−0.009^**^ (0.004)	−0.009^***^ (0.003)	−0.008^**^ (0.003)	−0.010^**^ (0.004)	−0.011^***^ (0.003)	−0.015^***^ (0.005)	−0.008^**^ (0.003)	−0.006^**^ (0.003)	−0.013^***^ (0.003)
Female labor force participation_t−1_	−0.031^***^ (0.003)	–	–	–	–	–	–	–	–
Log total population_t−1_	–	0.317^***^ (0.022)	–	–	–	–	–	–	–
Unemployment rate_t−1_	–	–	0.259^***^ (0.009)	–	–	–	–	–	–
Urban population_t−1_	–	–	–	0.100^***^ (0.003)	–	–	–	–	–
Transfers and subsidies_t−1_	–	–	–	–	−0.651^***^ (0.009)	–	–	–	–
Government Size Index_t−1_	–	–	–	–	–	1.313^***^ (0.047)	–	–	–
Labor market regulation_t−1_	–	–	–	–	–	–	0.206^***^ (0.030)	–	–
Overall globalization_t−1_	–	–	–	–	–	–	–	0.142^***^ (0.005)	–
Democracy/autocracy spectrum_t−1_	–	–	–	–	–	–	–	–	−0.127^***^ (0.010)
Observation	2,669	2,669	2,669	2,669	2,183	2,567	2,102	2,669	2,591
Countries	141	141	141	141	128	138	132	141	139
Wald Test [Probability]	11,126^***^ [0.000]	14,157^***^ [0.000]	11,409^***^ [0.000]	18,966^***^ [0.000]	13,739^***^ [0.000]	8,052^***^ [0.000]	12,943^***^ [0.000]	12,974^***^ [0.000]	10,149^***^ [0.000]

*The dependent variable is the post-tax Gini coefficient_t_. Intercept is included. The standard errors are in ().*

*** *p < 0.01 and*

** *p < 0.05*.

**Table 5:** Feasible General Least Squares (FGLS) estimations (non-OECD countries) (1996–2020).

**Sample**	**Non-OECD**	**Non-OECD**	**Non-OECD**	**Non-OECD**	**Non-OECD**	**Non-OECD**	**Non-OECD**	**Non-OECD**	**Non-OECD**
**Regressor**	**(1)**	**(2)**	**(3)**	**(4)**	**(5)**	**(6)**	**(7)**	**(8)**	**(9)**
Log Per Capita GDP_t−1_	2.352^***^ (0.060)	2.267^***^ (0.051)	2.292^***^ (0.055)	2.335^***^ (0.052)	2.425^***^ (0.081)	2.506^***^ (0.048)	2.089^***^ (0.071)	2.022^***^ (0.057)	2.601^***^ (0.061)
Age Dependency_t−1_	0.194^***^ (0.003)	0.213^***^ (0.003)	0.225^***^ (0.003)	0.219^***^ (0.003)	0.192^***^ (0.004)	0.240^***^ (0.003)	0.216^***^ (0.004)	0.229^***^ (0.003)	0.241^***^ (0.003)
WPDI*_*t*_*	−0.003^***^ (0.001)	−0.003^***^ (0.001)	−0.003^***^ (0.001)	−0.003^***^ (0.001)	−0.012^***^ (0.002)	−0.006^***^ (0.002)	−0.006^***^ (0.002)	−0.003^***^ (0.001)	−0.004^**^(0.002)
Female labor force participation_t−1_	−0.059^***^ (0.003)	–	–	–	–	–	–	–	–
Log total population_t−1_	–	0.125^***^ (0.026)	–	–	–	–	–	–	–
Unemployment rate_t−1_	–	–	0.086^***^ (0.008)	–	–	–	–	–	–
Urban population_t−1_	–	–	–	0.065^***^ (0.004)	–	–	–	–	–
Transfers and subsidies_t−1_	–	–	–	–	−0.415^***^ (0.017)	–	–	–	–
Government Size Index_t−1_	–	–	–	–	–	0.578^***^ (0.032)	–	–	–
Labor market regulation_t−1_	–	–	–	–	–	–	0.244^***^ (0.033)	–	–
Overall globalization_t−1_	–	–	–	–	–	–	–	0.058^***^ (0.005)	–
Democracy/autocracy spectrum_t−1_	–	–	–	–	–	–	–	–	−0.325^***^ (0.010)
Observation	1,889	1,889	1,889	1,889	1,446	1,826	1,373	1,889	1,850
Countries	107	107	107	107	94	104	98	107	105
Wald Test [Probability]	4,781^***^ [0.000]	5,331^***^ [0.000]	4,171^***^ [0.000]	5,303^***^ [0.000]	3,911^***^ [0.000]	7,162^***^ [0.000]	4,912^***^ [0.000]	4,690^***^ [0.000]	6,889^***^ [0.000]

*The dependent variable is the post-tax Gini coefficient_t_. Intercept is included. The standard errors are in ().*

*** *p < 0.01 and*

** *p < 0.05*.

**Table 6:** Feasible General Least Squares (FGLS) estimations (OECD countries) (1996–2020).

**Sample**	**OECD**	**OECD**	**OECD**	**OECD**	**OECD**	**OECD**	**OECD**	**OECD**	**OECD**
**Regressor**	**(1)**	**(2)**	**(3)**	**(4)**	**(5)**	**(6)**	**(7)**	**(8)**	**(9)**
Log per capita GDP_*t*−1_	−5.571^***^ (0.220)	−5.329^***^ (0.138)	−6.696^***^ (0.195)	−6.287^***^ (0.162)	−3.264^***^ (0.155)	−4.030^***^ (0.150)	−6.316^***^ (0.168)	−3.297^***^ (0.295)	−5.453^***^ (0.198)
Age dependency_*t*−1_	0.361^***^ (0.021)	0.379^***^ (0.015)	0.379^***^ (0.021)	0.263^***^ (0.023)	0.413^***^ (0.017)	0.388^***^ (0.016)	0.395^***^ (0.018)	0.287^***^ (0.023)	0.318^***^ (0.024)
WPDI_*t*_	0.072^***^ (0.021)	0.046^***^ (0.017)	0.048^***^ (0.018)	0.035^**^ (0.017)	0.009^***^ (0.003)	0.028^**^ (0.014)	0.021^**^ (0.009)	0.025^***^ (0.008)	0.075^***^ (0.020)
Female labor force participation_*t*−1_	−0.061^***^ (0.015)	–	–	–	–	–	–	–	–
Log total population_*t*−1_	–	2.017^***^ (0.069)	–	–	–	–	–	–	–
Unemployment rate_*t*−1_	–	–	0.198^***^ (0.026)	–	–	–	–	–	–
Urban population_*t*−1_	–	–	–	0.109^***^ (0.012)	–	–	–	–	–
Transfers and subsidies_*t*−1_	–	–	–	–	−0.480^***^ (0.012)	–	–	–	–
Government Size Index_**t**−1_	–	–	–	–	–	3.392^***^ (0.080)	–	–	–
Labor market regulation_**t**−1_	–	–	–	–	–	–	0.774^***^ (0.051)	–	–
Overall globalization_**t**−1_	–	–	–	–	–	–	–	0.259^***^ (0.020)	–
Democracy/autocracy spectrum_**t**−1_	–	–	–	–	–	–	–	–	−0.501^***^ (0.079)
Observation	780	780	780	780	737	741	729	780	741
Countries	34	34	34	34	34	34	34	34	34
Wald Test [Probability]	1,496^***^ [0.000]	5,056^***^ [0.000]	1,565^***^ [0.000]	1,448^***^ [0.000]	2,110^***^ [0.000]	4,024^***^ [0.000]	2,341^***^ [0.000]	1,373^***^ [0.000]	1,420^***^ [0.000]

*The dependent variable is the post-tax Gini coefficient_t_. Intercept is included. The standard errors are in ().*

*** *p < 0.01 and*

** *p < 0.05*.

Each additional control variable discussed in the Data section is included individually in the FGLS estimations. Tables 4–6 provide the estimated coefficient on the current WPDI. All results are in line with the benchmark estimations, and they are robust to the inclusion of nine additional control variables.

In the entire sample and the non-OECD countries' case, there are negative impacts of the WPDI on income inequality. The positive impact of the WPDI remains statistically significant in the case of the OECD countries.

More importantly, additional controls for potentially determining the after-tax income inequality, such as economic performance, labor market conditions, government size, globalization, and institutional quality, do not affect the statistical significance of the WPDI. Note that the importance of poverty is indirectly evaluated by the total unemployment rate, transfers and subsidies, and the high relevance of GDP per capita with these results. This evidence supports our main hypothesis that there are negative effects of pandemics on income inequality in developing economies, but the impact is positive in developed countries.

### Sensitivity Analyses

Table 7 provides the results of robustness checks by excluding the outliers from the dataset. Again, we consider the FGLS estimations of the lagged control model with the current WUI in Equation (3) for the post-tax income inequality using the data from 1996 to 2020.

**Table 7:** Sensitivity Analyses of the Feasible General Least Squares (FGLS) estimations (lagged controls) (1996–2019).

**Excluding**	**Indicator**	**All countries**	**Non-OECD**	**OECD**
Extreme observations of dependent variable	WPDI*_*t*_*	−0.009^***^ (0.003)	−0.005^***^ (0.001)	0.071^***^ (0.017)
Extreme observations of WPDI*_*t*_*	WPDI*_*t*_*	−0.008^***^ (0.003)	−0.005^***^ (0.002)	0.070^***^ (0.024)
LAC economies	WPDI*_*t*_*	−0.010^**^ (0.004)	−0.004^***^ (0.001)	0.057^**^ (0.023)
East Asia economies	WPDI*_*t*_*	−0.011^***^ (0.004)	−0.003^***^ (0.001)	0.055^***^ (0.019)

*The dependent variable is the post-tax Gini coefficient_t_. The standard errors are in ().*

*** *p < 0.01 and*

** *p < 0.05*.

Firstly, we exclude the extreme observations of the post-tax income inequality and the WPDI. Following Jha and Gozgor (47), extreme observation is defined as the values as more than two standard deviations away from the average. The results are robust to the exclusion of the extreme observations. Secondly, we individually exclude the observations of the Latin American and the Caribbean (LAC) as well as East Asian countries. We observe the baseline findings are robust to these sensitivity analyses. We conclude that observations from specific regions and extreme observations did not drive the baseline results.

Overall, various robustness checks confirm that pandemics uncertainty decreases the income inequality in the non-OECD countries, but it increases the income inequality in the OECD countries.

## Conclusion

This paper contributes to the literature by analyzing the effects of pandemics-related uncertainty on income inequality. We use a novel indicator of uncertainty—the World Pandemic Discussion Index (WPDI), introduced by Ahir et al. (26). This indicator is based on international discussions to measure the level of uncertainty related to pandemics at the country level. We find robust evidence that increases in the WPDI decrease the post-tax Gini coefficient in 107 non-OECD countries from 1996 to 2020. However, the FGLS estimations' findings indicate that the WPDI is positively associated with income inequality in 34 OECD countries. This finding is in line with Galletta and Giommoni (8) and Furceri et al. (25). Note that the evidence from Galletta and Giommoni (8) is based on the 1918 Influenza Pandemic and the case of Italy. We have enhanced their findings to 34 OECD economies and the globalization era (1996–2020). Furceri et al.'s (25) data is based on the unbalanced panel of 175 countries from 1961 to 2017. However, their method is based on the shocks of dummy variables for pandemics. We use the WPDI; therefore, we measure and compare the uncertainty due to the pandemic's magnitude over time across different countries.

Overall, our findings indicate that pandemics uncertainty is a significant determinant of income inequality, even though various macroeconomic variables and institutional quality controls are included. Furthermore, the findings suggest that pandemics have different effects on developed economies compared to developing economies. This evidence can be related to different business cycles, and it is in line with the results of the recent paper by Furceri et al. (25).

Finally, we need to enhance our knowledge on income inequality determinants considering the periods of uncertainty. Pandemics turned mandatory social isolation measures, which contributed to coming to an end several enterprises. However, it is important to note that our paper's findings are limited to the macro-level data. More precisely, identifying the exact mechanism relating pandemics-related uncertainty to income inequality requires research with the micro-level data. Thus, we can understand how an increase in pandemics uncertainty affects individual changes in income. At this stage, one can focus on the surveys or micro-level data further to understand the effects of pandemics uncertainty on income inequality. A Principal Component Analysis or the Bayesian Average techniques can be implemented for control variables' selection. Therefore, future studies can use micro-level data and different methods to capture the COVID-19 pandemic era in different countries to verify or reject our results.

## Data Availability Statement

Publicly available datasets were analyzed in this study. This data can be found here: https://worlduncertaintyindex.com/data.

## Author Contributions

TC: conceptualization, supervision, and writing-original draft preparation. GG: data curation, software, investigation, and writing-original draft preparation. CK: methodology, writing – reviewing and editing, and visualization. All authors contributed to the article and approved the submitted version.

## Conflict of Interest

The authors declare that the research was conducted in the absence of any commercial or financial relationships that could be construed as a potential conflict of interest.

## Appendix

**Table:**

One hundred and forty-one countries in the dataset.
Afghanistan, Albania, Algeria, Angola, Argentina, Armenia, Australia, Austria, Azerbaijan, Bangladesh, Belarus, Belgium, Benin, Bolivia, Bosnia and Herzegovina, Botswana, Brazil, Bulgaria, Burkina Faso, Burundi, Cambodia, Cameroon, Canada, Central African Republic, Chad, Chile, China, Colombia, Congo DR, Congo Republic, Costa Rica, Cote d'Ivoire, Croatia, Czech Republic, Denmark, Dominican Republic, Ecuador, Egypt, El Salvador, Ethiopia, Finland, France, Gabon, Gambia, Georgia Germany, Ghana, Greece, Guatemala, Guinea, Guinea-Bissau, Haiti, Honduras, Hong Kong, Hungary, India, Indonesia, Iran, Iraq, Ireland, Israel, Italy, Jamaica, Japan, Jordan, Kazakhstan, Kenya, Korea Republic, Kuwait, Kyrgyz Republic, Laos, Latvia, Lebanon, Lesotho, Liberia, Libya, Lithuania, Madagascar, Malawi, Malaysia, Mali, Mauritania, Mexico, Moldova, Mongolia, Morocco, Mozambique, Myanmar, Namibia, Nepal, Netherlands, New Zealand, Nicaragua, Niger, Nigeria, North Macedonia, Norway, Oman, Pakistan, Panama, Papua New Guinea, Paraguay, Peru, Philippines, Poland, Portugal, Qatar, Romania, Russia, Rwanda, Saudi Arabia, Senegal, Sierra Leone, Singapore, Slovak Republic, Slovenia, South Africa, Spain, Sri Lanka, Sudan, Sweden, Switzerland, Tajikistan, Tanzania, Thailand, Togo, Tunisia, Turkey, Turkmenistan, Uganda, Ukraine, United Arab Emirates, United Kingdom, United States, Uruguay, Uzbekistan, Venezuela, Vietnam, Yemen, Zambia, and Zimbabwe.
